# Crustal movement and strain distribution in East Asia revealed by GPS observations

**DOI:** 10.1038/s41598-019-53306-y

**Published:** 2019-11-14

**Authors:** Ming Hao, Yuhang Li, Wenquan Zhuang

**Affiliations:** The Second Monitoring and Application Center, China Earthquake Administration, Xi’an, China

**Keywords:** Geodynamics, Tectonics

## Abstract

East Asia is bounded by the Indian plate to the southwest and the Pacific and Philippine plates to the east, and has undergone complex tectonic evolution since ~55 Ma. In this study, we collect and process three sources of GPS datasets, including GPS observations, GPS positioning time series, and published GPS velocities, to derive unified velocity and strain rate fields for East Asia. We observed southward movement and arc-parallel extension along the Ryukyu Arc and propose that the maximum principal stress axis (striking NEE) in North China could be mainly induced by westward subduction of the Pacific plate and the southward movement of the Ryukyu Arc. The large EW-trending sinistral shear zone that bounds North China has been created by eastward movement of South China to the south and westward subduction of the Pacific plate to the north. GPS velocity profiles and strain rates also demonstrate that crustal deformation in mainland China is controlled by northeastward collision of the Indian plate into Eurasia and westward subduction of the Pacific and Philippine Sea plates beneath Eurasia. In particular, the India-Eurasia continental collision has the most extensive impact, which can reach as far as the southern Lake Baikal. The viscous behavior of the subducting Pacific slab also drives interseismic deformation of North China. The crustal deformation caused by Philippine oceanic subduction is small and is limited to the region between the southeast coast of mainland China and Taiwan island. However, the principal compressional strain around eastern Taiwan is the largest in the region.

## Introduction

**Figure 1:**
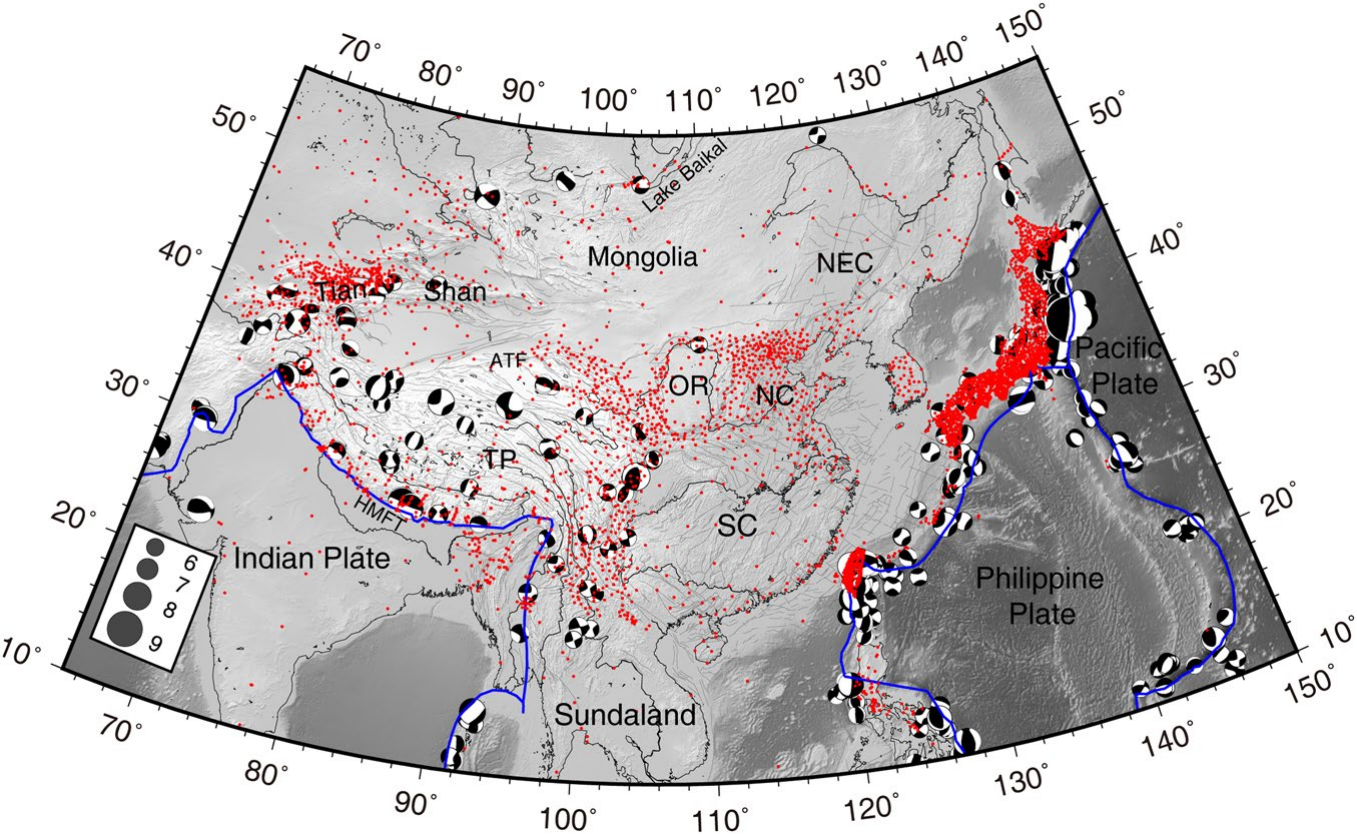
Distribution of GPS sites (red points) and earthquakes with magnitude ≥ M_w_ 6 in East Asia. Focal mechanism solutions are from the Global CMT catalogue (http://www.globalcmt.org/) from 1996~2015. Blue triangles define the South China reference frame. Blue curves are the primary plate boundaries from ref.^45^. Abbreviations are: Tibetan Plateau (TP), South China (SC), North China (NC), Northeast China (NEC), Ordos (OR), Himalayan Main Frontal Thrust (HMFT), and Altyn Tagh fault (ATF).

Cenozoic deformation of East Asia is complex due to the collision of India into Eurasia to the southwest and the Western Pacific and Philippine oceanic plates subducting under Eurasia to the east (Fig. 1). Since ~55 million years ago, the continued northward movement of India has uplifted the Himalayan orogen and Tibetan Plateau, and dominates the tectonic evolution of western mainland China^1–3^. In contrast, subduction of the Pacific and Philippine plates has created island arcs, marginal seas, continental rifting, and plays a crucial role in the tectonic stress in eastern China^3–5^.

The convergence between India and Asia causes intercontinental deformation over a large distances of up to ~3000 km, reaching as far as Lake Baikal^6^. Tomographic images also indicate that the dynamic processes of the Western Pacific and East Asia are responsible for the formation of continental rifts and active intraplate volcanoes in Northeast China^7,8^. However, the dynamic sources of tectonic motion in East Asia remain elusive. Some have proposed that the tectonics of East Asia can be entirely related to the India-Eurasia collision^1,9^, while others emphasize that the stress field of East Asia is controlled by the combined convergence of the Indian, Pacific, and Philippine plates^10,11^. The advent of GPS has provided an accurate and efficient approach for measuring regional and large scale crustal deformation, which are crucial in the attempt to constrain the dynamic processes affecting East Asia.

Despite extensive previous studies that have provided GPS-derived regional crustal motions for regions such as the Tibetan Plateau, Tian Shan, western Pacific subduction zone, and even a sparse velocity field for all of Asia^2,12–17^, a lack of a high resolution and accurate GPS velocity field has prevented the elucidation of crustal motion across the whole of East Asia. Our primary purpose in this study is to generate present-day unified GPS velocity and strain rate fields for East Asia, in order to analyze the patterns of crustal movement across key regions around East Asia. We also discuss reasons for the tectonic stress field direction in North China and compare the influences of continental collision and oceanic subduction on the overall stress field. The GPS velocity field generated and discussed herein is helpful for understanding various regional kinematics and geodynamics that drive continental deformation, and is useful for maintaining modernized geodetic datum.

### GPS data

We assembled three sources of GPS datasets to derive a geodetically consistent crustal velocity field for East Asia. The first dataset is raw GPS observations measured in mainland China. The second is continuous GPS time series data available from public online websites (http://www.gsi.go.jp/, Geospatial Information Authority of Japan, GSI, and http://tec.earth.sinica.edu.tw/, Taiwan Earthquake Research Center, TEC), which provide GPS data for the Western Pacific and Philippine subduction zones. The third one is GPS velocities from existing literature, which mainly focus on the India-Asia collision regime.

The GPS data collected and measured in mainland China are from the Crustal Movement Observation Network of China (CMONOC). These GPS stations were surveyed in 1998, 1999, 2001, 2004, and 2007. We did not use any post-2007 data in order to avoid effects of the 2008 M_w_ 7.9 Wenchuan and 2011 M_w_ 9.0 Tohoku earthquakes. The GAMIT software package^18^ was employed to get daily loosely constrained solutions for satellite orbits and coordinates of regional stations. Approximately 100 evenly distributed ITRF core GPS sites were also processed using the GAMIT software. The VMF1 tropospheric mapping function was used to calculate the wet and hydrostatic zenith troposhperic delays. The GLOBK software^19^ was used to estimate daily positions and uncertainties by combining daily regional and global solutions, and then the daily free network solutions was transformed into ITRF2008^20^. The GPS sites used to define the global ITRF2008 are showed in Fig. S1 in the Supplementary Material. Due to the coseismic deformation of the 2004 M_w_ 9.1 Sumatra earthquake, we also estimated offsets by using the coseismic slip model to correct the time series. Section A2 and Fig. S2 in the Supplementary Material show the coseismic displacements of the campaign mode GPS stations. We considered the postseismic displacements of this earthquake to be negligible, for the purposes of this study. Then we used a linear function to fit the GPS time series, in order to estimate secular velocity rates with respect to ITRF2008.

The continuous GPS positioning time series used span 1996~2015 and were provided by GSI and TEC. In order to mitigate the seasonal variations in these GPS time series, we only estimated velocities for time series that were at least 2.5 years long. We preferred ruling out parts of GPS time series that also demonstrated prominent transient deformation rather than employing proper models to correct them, because modeling transient deformation, including postseismic deformation, slow slip, or other non-linear processes, can introduce a large number of model dependencies^15^. Finally, we applied a linear trend and a seasonal variation to fit the GPS time series and infer interseismic velocity. It is well known that errors in daily GPS position estimates are time correlated^21^. Using a purely white noise model would cause low velocity uncertainties. Therefore, we employed the Maximum Likelihood Estimation technique using the CATS software^21^ to estimate velocity uncertainties, with the assumption that the error can be categorized by a power law noise model. More details regarding our estimation procedure are included in section A3 of the Supplementary Material.

The GPS velocities derived from previously published studies are mostly distributed in the India-Asia collision regions. Table 1 lists previous studies used in GPS compilation and the regions our study concerned. These secular velocities were originally determined with respect to different global (e.g., ITRF2000, ITRF2005 and ITRF2008) or regional reference frames (e.g., Eurasian and Indian plates).

**Table 1:** Published studies used in GPS compilation.

Studies	Regions concerned	Reference frame
ref.^13^	Tian Shan	ITRF2005
ref.^15^	South Korea	ITRF2008
ref.^46^	Nepal	ITRF2000
ref.^47^	Thailand	Sundaland block
ref.^48^	India and southern Tibet	ITRF2000
ref.^49^	Northern Myanmar	ITRF2000
ref.^50^	Pamir and Hindu Kush	ITRF2005
ref.^51^	Southeastern Russia	Eurasian plate
ref.^52^	Southeastern Russia	ITRF2008
ref.^53^	Baikal–Mongolia	Eurasian plate
ref.^54^	Nepal	ITRF2005
ref.^55^	Northwestern Vietnam	ITRF2008
ref.^56^	Pamir and Hindu Kush	ITRF2008
ref.^57^	Kashmir	Indian plate
ref.^58^	Myanmar	ITRF2005
ref.^59^	India and Myanmar	ITRF2008
ref.^60^	Kashmir	ITRF2008
ref.^23^	India and Southern Tibet	Indian plate

Because we would like to obtain GPS movement across mainland China and its adjacent regions, a regional stable reference frame within mainland China is suitable. Velocities for 62 GPS stations located on the stable South China block were selected to estimate the Euler pole by applying constraints that minimized motions within the interior of the South China block. We removed potential outliers from the velocities through an iterative procedure: we removed stations with the largest post-fit residual and re-estimated the angular velocity until all the post-fit residuals were less than 2 mm/yr. Finally velocities for 50 stations (blue triangles, Fig. 1) were used to define the South China block. The estimated pole position of the South China block is located at 53.420 ± 1.275°N, −101.102 ± 1.139°W, with a rotation rate of 0.311 ± 0.006°/Ma. Section A4 in Supplementary shows the detailed procedure. Then we transformed the velocity field of mainland China into a regional reference frame with respect to the South China block by using a rigid block rotation.

**Figure 2:**
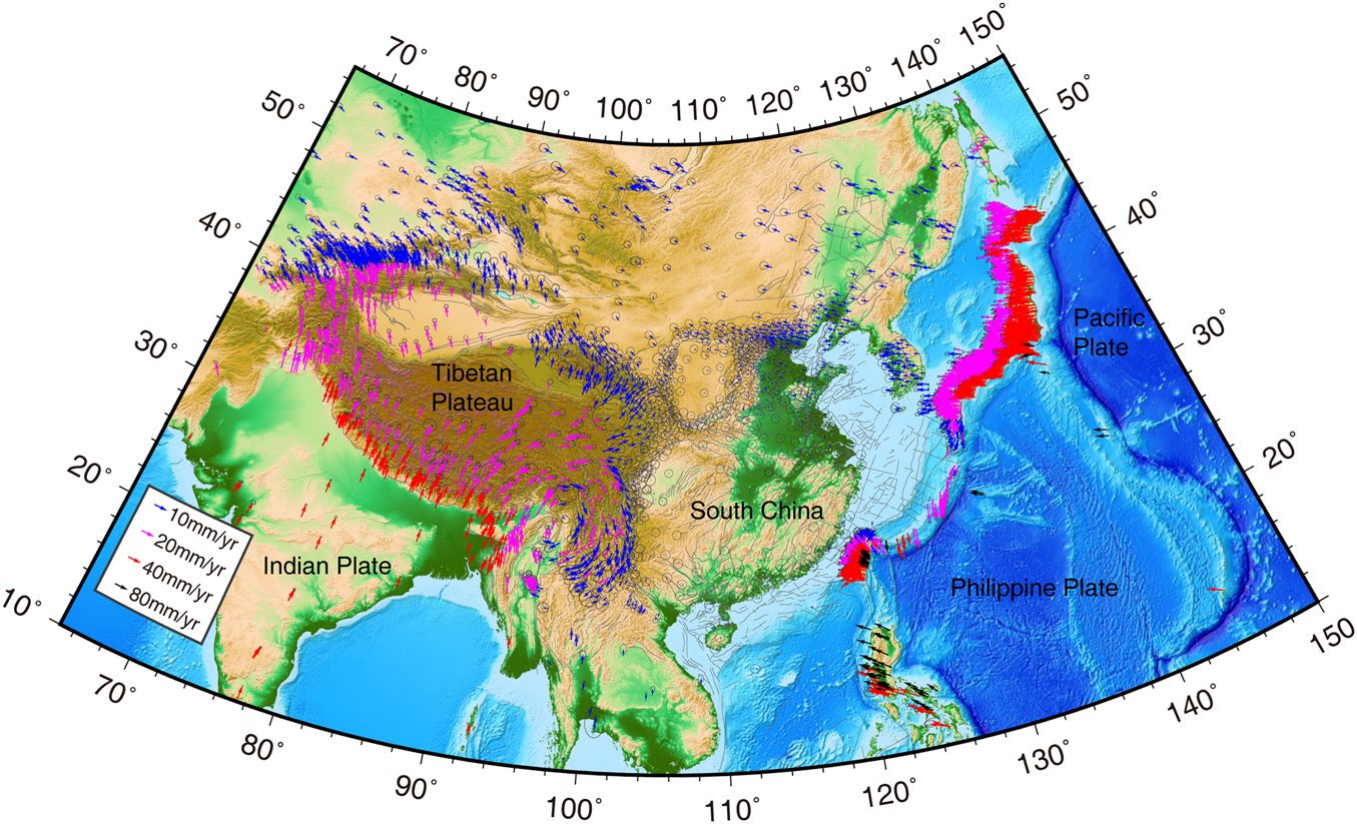
GPS horizontal velocity field within East Asia, with respect to a stable South China. The thin gray curves indicate major active faults in the late Quaternary. Error ellipses are 95% confidence intervals. GPS velocities for the western Pacific area are shown in Fig. 3 for clarity.

We chose GPS velocities within mainland China as the reference data set, and velocities obtained from other published papers and estimated from GPS time series were first transformed to this regional reference frame. Here, we employed the two-dimensional Helmert transformation (east and north velocities). The four parameters were determined (two translations, one scaling and one rotation about the Z-axis) for our data and other velocities to be transformed with at least two identical points, using the least squares method. More than two identical points, along with those with sums of post-fit residual squares, were used to estimate the transformation parameters. The post-fit residuals following the Helmert transformation were generally less than 1.0 and 1.0 mm/yr in the eastward and northward velocities of identical points, respectively. We present the GPS velocity field for East Asia with respect to a stable South China block (Fig. 2). Section A6 in the Supplementary Material provides the estimated GPS rates and errors.

We also calculated a strain rate field from GPS velocities using the method proposed by ref.^22^. This technique employs spherical wavelet-based multi-scale decomposition to calculate a continuous velocity field from discrete GPS observations. The coefficients of the spherical wavelets are then used to directly compute strain rates^23^. The advantages of this technique are that (1) the velocity field is decomposed into multiple scales at all stations explicitly and consistently, and (2) the minimum scale of the estimated velocity field for a particular location is controlled by the local spatial density of observations.

## Results

### Velocity field

The horizontal velocity field (Fig. 2) shows first-order deformation in East Asia. To the southwest, the Indian plate moves northward at rates of ~37 mm/year. The Tibetan Plateau experiences NS shortening and EW extension. The Tian Shan orogen, especially the western section, absorbs a large amount of crustal shortening, with rates of 15~18 mm/yr. With respect to South China, the east side of the eastern Himalayan syntaxis (EHS) shows clockwise rotation, while its west side demonstrates movement in the NNE-NE direction.

**Figure 3:**
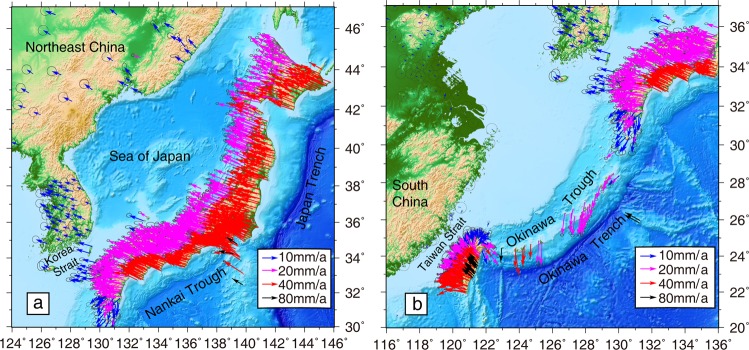
Horizontal GPS velocity field of the western Pacific. (**a**) Velocities across Japan and Northeast China. (**b**) Velocities across the Ryukyu Arc and Taiwan island. Error ellipses are 95% confidence intervals.

To the east, due to the subduction of the Pacific plate, northern Japan moves westward. Velocity rates are ~40 mm/yr along the east coast of Japan and decrease to ~20 mm/yr along the coast of the Japan Sea (Fig. 3a). Crossing over the Japan Sea, velocities in southeast Russia and Northeast China trend to the NW, with rates of 6~8 mm/yr. Further south, there is a velocity gradient trending NWW, which is located in the Zhangjiakou-Bohai Seismic Zone (ZBSZ) and forms the northern boundary of North China^24^. Velocity rates decrease from 4~5 mm/yr in the north to 1~2 mm/yr in the south, showing sinistral slip motion.

To the southeast, the Philippine plate subducts NW under the Ryukyu Arc with a rate of ~80 mm/yr (Fig. 3b). The GPS velocities in the Ryukyu Arc increase from 14 mm/yr in the north to 40 mm/yr in the south, while the velocity directions gradually shift from southwest to south. Eastern Taiwan undergoes significant contraction, at rates of approximately 20 mm/yr. Further south, the northern Luzon arc moves NW at a rate of ~80 mm/yr.

### Strain rate fields

**Figure 4:**
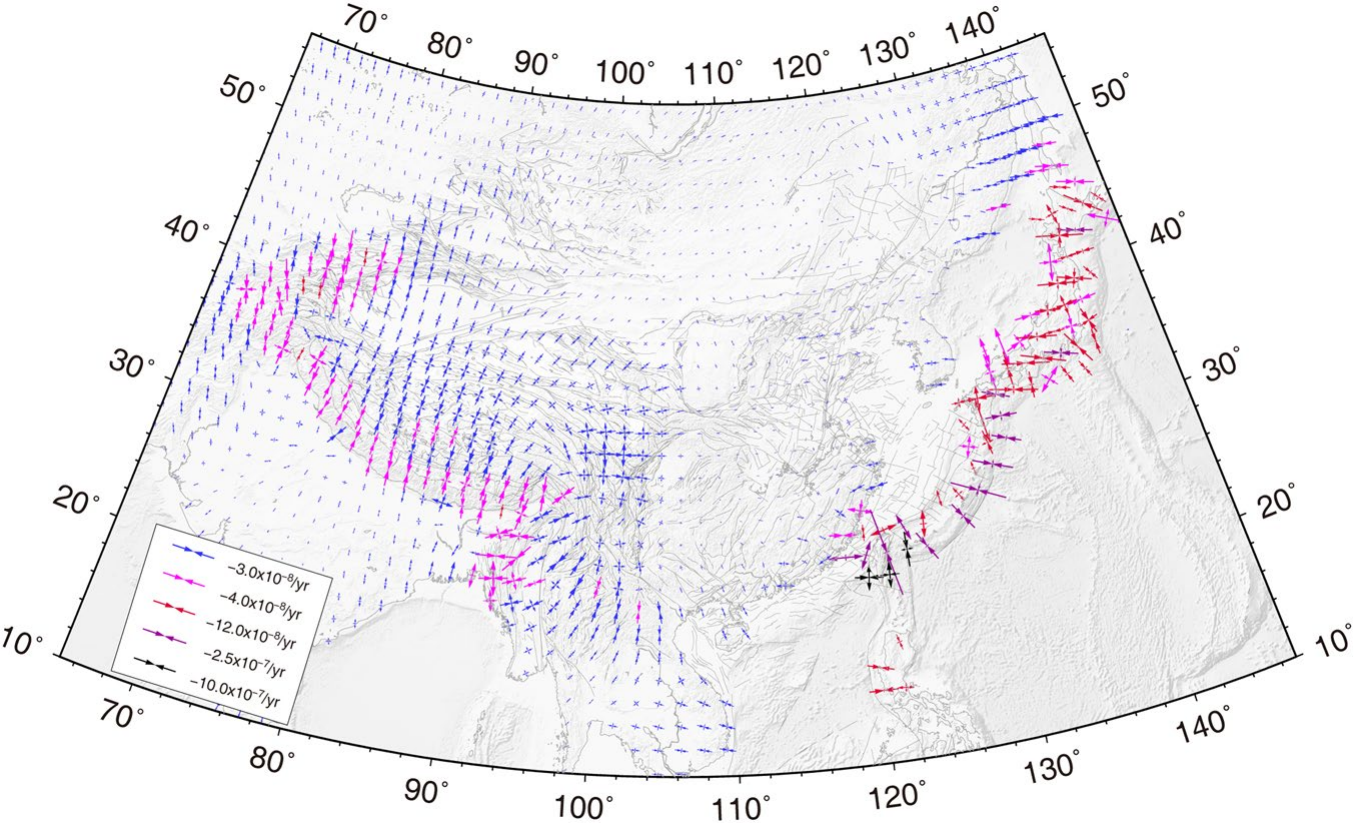
Principal strain rate field for East Asia.

The key feature of the principal strain rate field (Fig. 4) is that the contractional strain rates around the three plate boundary zones are the most prominent. Especially the Philippine subduction zone, which generates the highest compressive strain rates of greater than 100 × 10^−8^/yr in a NWW direction located along the east coast of Taiwan. The principal compressive strain rate along the east coast of Japan caused by Pacific plate subduction is also higher than along the Himalayan Main Frontal Thrust, which is induced by the convergence of the Indian plate. The western Tian Shan also has high compressive strain rates, as does the Himalayan Main Frontal Thrust. The strain rate across the Tibtean Plateau is consistent with ref.^25^, excluding the magnitudes of compressive strain rate in Nepal. The compressive strain rate^25^ in this region is larger than ours. By comparing the two GPS velocity fields, we found our GPS velocities are more than ref.^25^ in the Himalayan orogen and the Indian plate. However, our derived GPS strain rate is similar to ref.^26^ in the Himalayan orogen.

**Figure 5:**
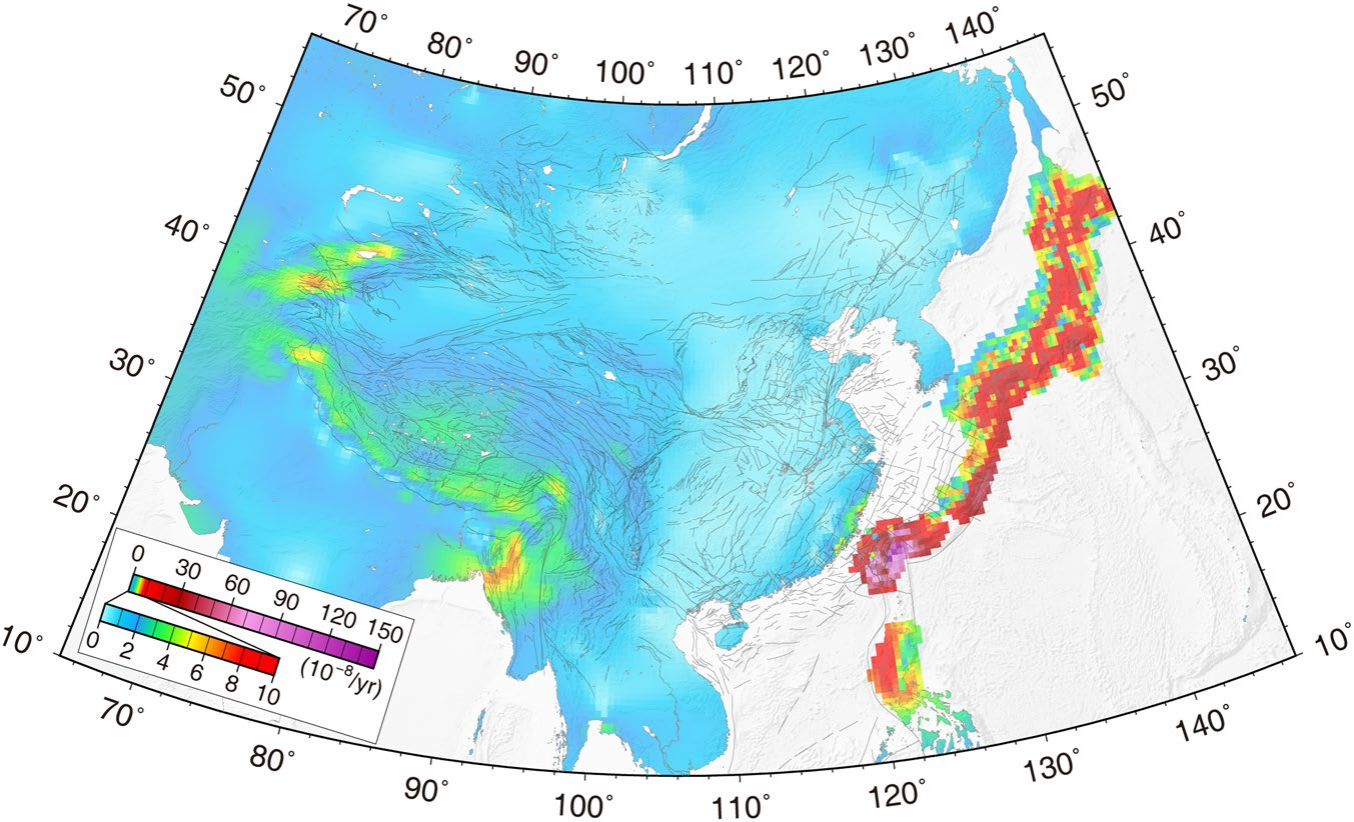
Maximum shear strain rate field for East Asia.

Figure 5 shows the maximum shear strain rates in East Asia. We observed that maximum shear strain rates of 10 × 10^−7^/yr, 3 × 10^−7^/yr, and 1 × 10^−7^/yr around Taiwan, the Ryukyu Arc, and Japan, respectively. The Indo-Burmese range, eastern and western Himalayan syntaxises, and Sagaing fault also have relatively high shear strain rates.

## Discussion

### Southward movement along the ryukyu arc

As shown in Fig. 3, crustal deformation in Japan shows westward movement along the Japan Trench and NWW movement along the Nankai Trough, which are both attributed to the subduction of the Pacific plate. However, further south the velocity vectors are in a S-SSW direction along the Ryukyu Arc. The geophysical, geological, and GPS data show that there is substantial contraction along the Nankai Trough, but no contraction along the Ryukyu Trench^27^. Moment tensor solutions also show that arc-parallel extension exists throughout the Ryukyu Arc region, excluding its northeastern edge^28^.

GPS-derived strain rates for the Ryukyu Arc show arc-perpendicular contraction and arc-parallel extension (Fig. 4). ref.^28^ proposed that the arc-parallel extension might not be due to the effects of oblique subduction alone; the back-arc opening process could play a crucial role in this arc-parallel extension^28–30^. Tectonic morphology and geophysical soundings reveal that en echelon central depressions are distributed along the axis of the Okinawa Trough. Both sides of the depressions contain normal faults extending tens of kilometers, indicating that these depressions are grabens. This implies that the trough has been in a state of extension^29,31^. Independently calculated opening rates for the Okinawa Trough are 10~50 mm/yr^29^, consistent with our GPS velocity rates of 14~40 mm/yr for the region.

### Geodynamics of stress orientation and strain distribution in north china

North China is bounded by the Pacific plate to the east, the Ordos block to the west, stable South China to the south, and Northeast China (belongs to the Amurian block) to the north. ref.^32^ used focal mechanisms, fault striations inferred from Quaternary fault slip measurements, stress relief, and hydraulic fracturing data to estimate the tectonic stress field for China. Their results indicated that the maximum principal stress axis strikes NEE in North China. However, a compressive stress orientation of NEE cannot be attributed only to the northward collision of the Indian plate and the Pacific plate westward subduction^10^, since the Pacific plate subduction in a NWW direction seems unlikely to generate such a large deflected angle.

Our GPS velocity field shows that Japan moves westward due to Pacific plate subduction, while the Ryukyu Arc moves southward as a result of Okinawa Trough extension (Fig. 3). Therefore, the westward subduction of the Pacific plate and southward movement along the Ryukyu Arc can generate a principal compressive stress in the NE-SW direction, resulting in the maximum principal stress axis striking NEE in North China.

**Figure 6:**
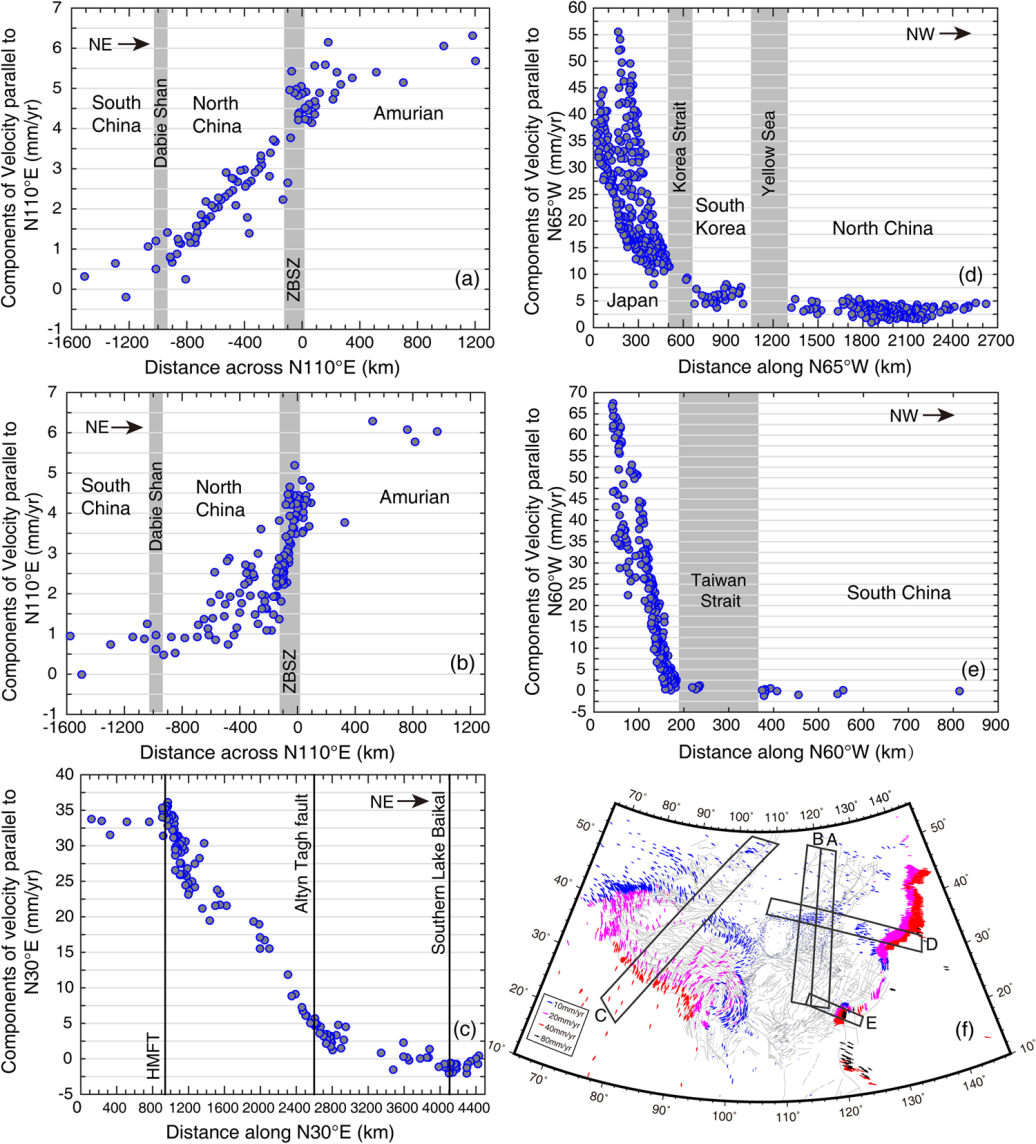
GPS velocity profiles across the convergence zones of the Indian, Western Pacific, Philippine plates and North China. The westward and northward GPS velocity components are positive. (**a**,**b**) Show GPS velocity components parallel to N110°E across eastern and western North China, respectively (A and B in **f**). (**c**) Shows GPS velocity components parallel to N30°E across the Himalayan thrust zone and Tibet (C in **f**). (**d**) Shows GPS velocity components parallel to N65°W across the Japan subduction zone and North China (D in **f**). (**e**) Shows GPS velocity components parallel to N60°W across the Taiwan plate boundary zone (E in **f**). (**f**) Shows the approximate locations of the profiles.

Components of GPS velocity profiles (Fig. 6a,b) parallel to the ZBSZ across North China show that the ZBSZ has a sinistral slip rate of 3 mm/yr. The GPS velocity field, with respect to South China (Fig. 2), demonstrates that the directions of GPS velocities in Northeast China are primarily to the NWW and the magnitudes decrease gradually from east to west. Consistently, the magnitudes of principal compressive strain rates in Northeast China also decrease from east to west and their azimuths change from EW to NE-SW. This suggests that the westward motion of the Pacific plate plays a crucial role in driving NW-ward movement in Northeast China.

The kinematics of the ZBSZ may also be caused by westward motion of the Pacific plate. Through analyzing GPS velocity profiles, ref.^24^ found that interseismic deformation in North China absorbs left-lateral shear of 6.0 mm/yr over an ~1100 km distance. They proposed a model that an EW-trending sinistral shear zone (the north and south boundaries being the ZBSZ and Dabie Shan, respectively) bounds a group of NEE-trending dextral strike-slip faults within North China^33^. ref.^24^ suggests that the sinistral shear strain across North China related to the stable Amurian block is created by eastward movement of South China, which is driven by eastward expansion of the Tibetan Plateau to the south. Additionally, our results indicate that the sinistral shear across North China could be caused by NW-ward movement of Northeast China caused by westward subduction of the Pacific plate to the north.

### Kinematics influence of the Indian plate colliding with eurasia

As mentioned previously, the dynamic sources of tectonic deformation in East Asia are still in question. Some believe that the tectonic features of East Asia are mainly attributable to the collision of the Indian plate with Eurasia^1,9^, while others suggest that the stress field of East Asia is governed by the combined influences of the Indian, Pacific, and Philippine plates converging with the Eurasian plate^10,11^.

Our unified GPS velocity and strain rate fields for East Asia can shed new light on the range of crustal deformation caused by the plates colliding with Asia. In order to reveal crustal deformation along the direction of subduction for each plate explicitly, we constructed three velocity profiles across the Himalayan thrust zone (Fig. 6c), Japan subduction zone (Fig. 6d), and Taiwan plate boundary zone (Fig. 6e). The approximate profile locations are shown in Fig. 6f.

Figure 6c shows the velocity components parallel to N30°E, from the Indian plate in the south to Lake Baikal in the north. We observe significant shortening rates of ~30 mm/yr along N30°E across the Tibetan Plateau between the HMFT and the Altyn Tagh fault, over a distance of 1700 km. Further north, the N30°E components of velocity gradually decrease from 5 mm/yr on the Altyn Tagh to −2 mm/yr on southern Lake Baikal, over a distance of 1500 km. The azimuths of the principal compressive strain rates change from N in the HMFT, to NNE in the Tibetan Plateau, and to NE in Mongolia and southern Lake Baikal. The magnitudes of principal compressive strain rates decrease from 2 to 0.1 × 10^−8^/yr along this profile (Fig. 4).

Geophysical studies reveal that the Indian plate is underthrusting central Tibet at a low angle and reaches north to the Bangong–Nujiang Suture (~500 km north of the HMFT)^5,34,35^. However, the gradual decrease in velocities and compressive strain rates in the NEE-NE direction suggests that the lithospheric deformation generated by the convergence of the Indian plate has extended ~3000 km away from the HMFT on the Earth’s surface^1^.

**Figure 7:**
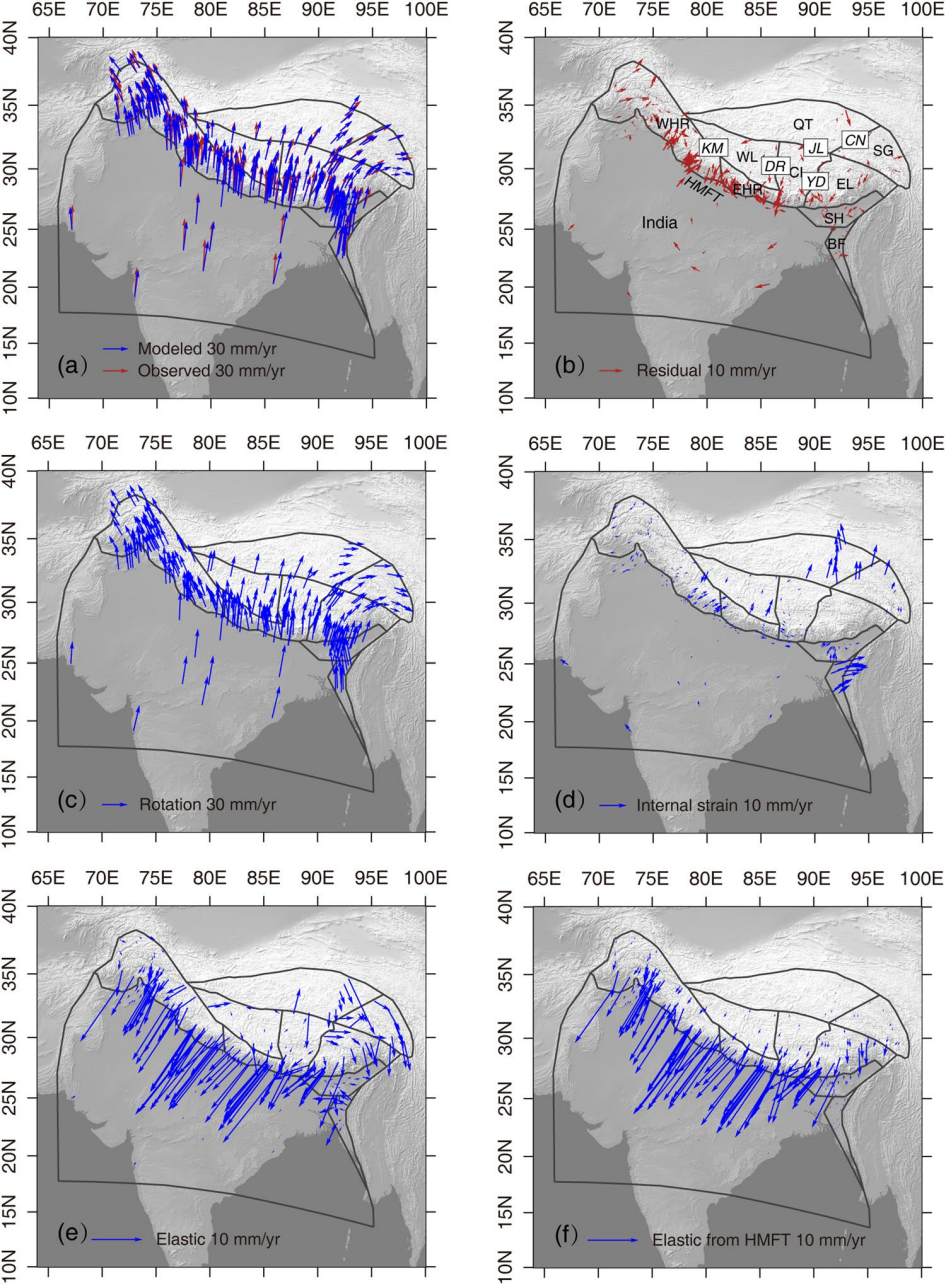
GPS velocities across the HMFT modeled by the Block theory. (**a**) Observed and modeled GPS velocities. (**b**) Postfit residual. (**c**) GPS velocities result from block rotation. (**d**) GPS velocities result from internal block strain. (**e**) GPS velocities result from elastic strain accumulation from locking faults. (**f**) GPS velocities result from elastic strain accumulation from locking on the HMFT. The blocks are labeled in (**b**) are West Himalayan Range (WHR), East Himalayan Range (EHR), West Lhasa (WL), Central Lhasa (CL), East Lhasa (EL), Qiangtang (QT), and Songpan-Ganzi (SG). Major faults labeled in (**b**) are the Karakorum (KM), Jali (JL), Yadong (YD), Dangreyongcuo(DR), and Cuonaanduo(CN).

We employed spherical linear block model^36,37^ to investigate the crustal movement due to elastic strain accumulation from locking on the HMFT. The block geometry, dip angle and locking depth are determined from ref.^36^. The block model result suggests that crustal deformation result from elastic strain accumulation on the locking HMFT is confined within south of the Jali fault, and directs southward (Fig. 7(f)). The estimated slip rates along faults are provided in the Supplementary Material. ref.^36^ also used block model constrained by GPS velocities to infer the extent to which relative motion of the Indian and Asian plates is partitioned between localized slip on major faults and distributed deformation in the Tibetan Plateau. Their results suggest that microplate rotation, interseismic elastic strain accumulation, and fault slip on the boundaries of microplates account for surface movement in the Tibetan Plateau. Internal block deformation occurs within some micro-plate, such as the Himalayan Range, Jiali, and west-central plateau blocks. Therefore, the observed northward movement within Tibet and further north is mainly from block rotation and internal strain, which is driven by boundary forces possibly^38^.

### Kinematics influence of the pacific plate subducting under eurasia

Figure 6d shows the velocity components parallel to N65°W, from southern Japan through Korea and the ZBSZ, to the north side of the Ordos block. In general, velocity components that are parallel to N65°W decay with distance from the plate boundary, but the velocity gradient across southern Japan is the largest. Although there are no GPS sites in the Korea Strait or the Yellow Sea, we obtain NW-SE contraction rates of about 4 and 2 mm/yr, respectively, from this velocity profile. Along the ZBSZ in North China, the change in velocity components towards the west is insignificant. The principal compressive strain rates decrease from 15 × 10^−8^/yr near central southern Japan, to 0.01 × 10^−8^/yr near the northern side of the Ordos block, and the corresponding azimuths rotate from E-W to NEE-SWW (Fig. 4).

Components of velocity that are parallel to N110°E across eastern North China (Fig. 6a) suggest that eastern North China, from the Dabie Shan to the ZBSZ, experiences a pronounced sinistral shear, while velocity components parallel to N110°E across western North China (Fig. 6b) suggest that sinistral shear strain is primarily concentrated in the vicinity of the ZBSZ, not the entire North China region. Therefore, the influence of Pacific plate subduction on North China weakens from east to west, and is mainly distributed in the eastern region.

Block model constrained by GPS velocity was used to estimate plate movement, fault slip rates and interplate coupling^37^. However, movement due to elastic strain accumulation from locking on the subduction zone interfaces is NEE, which is opposite to the subduction direction of the Pacific plate. The westward motion of Japan that gradually decreases from east to west reflects the E-W contractional strain built up by inter-plate coupling^39^. Interseismic deformation induced by subduction is controlled by viscous behavior of the mantle^40^. Tomographic images show that the subducting Pacific slab is represented by a prominent high-velocity zone, and the flat Pacific slab extends from Japan to Beijing, China, over 2300 km, at a depth of 660 km^5,7,41,42^. This suggests that Pacific subduction beneath Asia is the major cause for active tectonics and mantle dynamics in a broad region between the Japan Trench and East Asia, rather than the India-Asia collision^7^. Driving forces from Pacific plate subduction can play a key role in crustal movement decaying from east to west in eastern mainland China.

### Kinematics influence of the philippine plate subducting under eurasia

Figure 6e shows the velocity components that are parallel to N60°W from the east coast of Taiwan, through the Taiwan Strait, to South China. From east to west, the velocity components parallel to N60°W demonstrate large convergence rates of ~50 mm/yr across Taiwan, but only a small velocity gradient across South China. The strain rates along the profile show that eastern Taiwan is experiencing NW-SE intensive contraction, with maximum rates of 110 × 10^−8^/yr, and the southeast coast of South China, across the Taiwan Strait, undergoes E-W contraction with maximum contraction rate of 3.5 × 10^−8^/yr. The principal strain rates are insignificant in South China.

Taiwan is situated on a plate boundary zone between the Eurasian and Philippine plates. The Philippine plate subducts beneath the Eurasian plate along the Ryukyu Arc in the east, while the Eurasian plate subducts eastward beneath the Philippine plate. P- and S-wave velocity structures and *P*_*n*_ tomography show high velocity anomalies beneath the Ryukyu and Luzon arcs at depths ranging from 100~200 km, and the anomalies related to the Eurasian and Philippine slabs appear to be connected^34,43^. ref.^43^ proposed that the origin of the island of Taiwan was induced by collision at the edge point of the two subduction zones, resulting in prominent shortening and strong stresses confined to the eastern Taiwan region. Comparing the geophysics, geology, and GPS data with the theoretical results calculated from the Euler parameter of the Philippine Sea plate, ref.^27^ proposed that the collision of the Eurasian and Philippine plates in the area of Taiwan between 21.5°N and 24.2°N causes strong compression in the southeast part of the Chinese continent, but with a limited scope.

### Range of kinematics influence of three plates colliding with eurasia

The Indian-Eurasian continental collision and continued northward movement of the Indian plate are responsible for crustal deformation that reaches as far as southern Lake Baikal. The Pacific plate subducting under Eurasia may affect tectonic movement in an area from the Japan subduction zone to North China, while the northwestward subduction of the Philippine plate influences the surface deformation of the southeastern coast of the Chinese continent. The northeastward collision of the Indian plate with Asia and the westward subduction of the Pacific and Philippine Sea plates may have played a significant role in the formation and tectonic evolution of mainland China^5^.

In this paper, we emphasize that the first-order drive forces resulting from India-Eurasia collision and subduction of the Pacific and Philippine plates beneath Eurasia control the deformation of mainland China. The regional tectonic deformation of China’s continent is also characterized by the coupling of rigid block movement and continuous deformation. Regions of high rigidity behave as rigid block-like movement, while those of low rigidity are dominated by continuous deformation. Rheological flow in the lower crust and upper mantle plays an important role in controlling deformation of the upper crust^44^. Using thin sheet model, ref.^38^ believed tectonic deformation across Asia is driven by the balance of boundary forces and buoyancy stresses generated by gravitational potential energy gradients. Their result shows that deformation in contractional regions (such as Himalayas, Tibet and Tian Shan) is well result from boundary forces with strong coupling at the India-Eurasia collision zone. But southeastward movement of North and South China requires buoyancy stresses across the Pacific and Philippine subductions.

The GPS velocity filed used in ref.^38^ is respect to Eurasia, so the GPS velocities observed in South and North China move to east and southeastward. Therefore, buoyancy forces are needed to resist to boundary forces arising from subduction of oceanic plates beneath Asia. However, our GPS-derived velocities are respect to South China, a stable regional block within mainland China, and North China moves northwestward which is consistent with the direction of oceanic plates’ subduction. Whether buoyancy forces still required needs further simulation constrained with our GPS velocity filed across East Asia.

## Conclusions

We used GPS measurements to determine the present day unified GPS velocity field for East Asia. The velocities and calculated strain rates show first-order crustal movement induced by the Indian plate penetrating into Eurasia, and the Western Pacific and Philippine Sea plates subducting beneath Eurasia. Our GPS velocity results demonstrate southward movement and arc-parallel extension along the Ryukyu Arc, which may be attributed to back-arc extension. We propose that westward movement of the Pacific plate and southward movement along the Ryukyu Arc could produce a principal compressive stress in the NE-SW direction, resulting in a maximum principal stress axis striking NEE in North China. The large EW-trending sinistral shear zone bounding North China could have possibly been created by eastward movement of South China to the south, and westward subduction of the Pacific plate to the north.

Using GPS velocity profiles along the subduction direction of the Indian, Pacific and Philippine plates separately, we discussed the range of kinematics influence of these subduction boundaries. Our results indicate that tectonic movement in mainland China is controlled by the northeastward collision of the Indian plate and westward subduction of the Pacific and Philippine Sea plates beneath Asia. The Indian-Eurasian continental collision has much more extensive impact than the Pacific and Philippine oceanic plates subducting beneath Eurasia, but the contractional strain caused by the Philippine subduction zone is the largest.

## Supplementary information

Supplementary material

Dataset 1
