# Supplementary material for: Crustal movement and strain distribution in East Asia revealed by GPS observations
Source: Sci Rep. 2019 Nov 14;9:16797. doi: 10.1038/s41598-019-53306-y (PMC6856154; doi:10.1038/s41598-019-53306-y)
Supplement: Supplementary file 1 — Supplementary material [file 41598_2019_53306_MOESM1_ESM.pdf]

Crustal movement and strain distribution in East Asia revealed by GPS observations

Ming Hao, Yuhang Li, Wenquan Zhuang

The Second Monitoring and Application Center, China Earthquake Administration

A1. GPS sites used to define the global ITRF2008

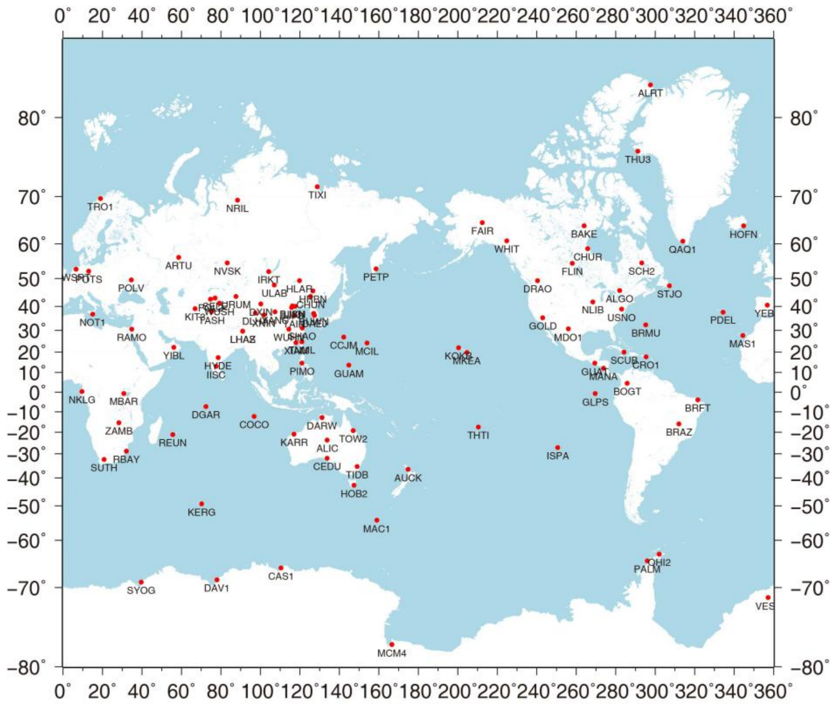

Figure S1. GPS sites used to define ITRF2008

A2. Coseismic displacements following large earthquakes

Three large earthquakes, the 2001 M_w 7.8 Kokoxili earthquake in Tibet, 2004 M_w 9.1 Sumatra-Andaman earthquake and 2005 M_w 8.7 Nias-Simeulue earthquake in the Sumatra subduction zone occurred between 1998 and 2007, and could affect the GPS data measured in mainland China.

Wan et al. (2008) provide the coseismic displacements of the 2001 Kokoxili earthquake at 34 GPS sites located within 350 km from surface rupture. We used these coseismic offsets to correct GPS time series directly.

For the 2004 Sumatra-Andaman earthquake we used the coseismic slip distribution provided by Chlieh et al. (2007) in a 3-D heterogeneous spherical earth model (Fu and Sun, 2008) to calculate the coseismic deformation field in mainland China (Figure S1). Figure S1 shows the coseismic offsets greater than 3 mm. We can see significant coseismic displacements are recognized at GPS sites in the southeastern Tibetan Plateau and South China.

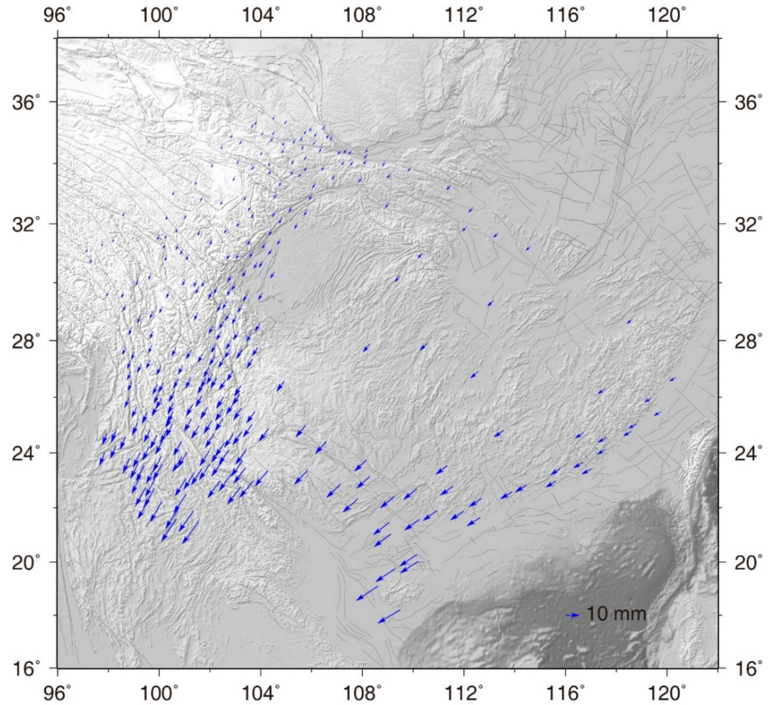

Figure S2. Modeled coseismic displacements following the Sumatra earthquake

We also used the slip distribution model provided by Konca et al. (2007) to estimate the coseismic displacements of the 2005 Nias-Simeulue earthquake. Our result shows that the coseismic displacements at GPS sites in mainland China are less than 1 mm which can be negligible.

A3. Method of velocities' estimation from continuous GPS time series

We chose GPS time series spanning longer than 2.5 years to estimate horizontal velocities. We preferred ruling out parts of GPS time series that demonstrate prominent transient deformation rather than employing proper models to correct them, because modeling non-linear transient deformation can introduce a lot of model dependencies (Kreemer et al, 2014). Figure S2 shows examples of GPS time series fit by linear trend, annual and semi-annual variations.

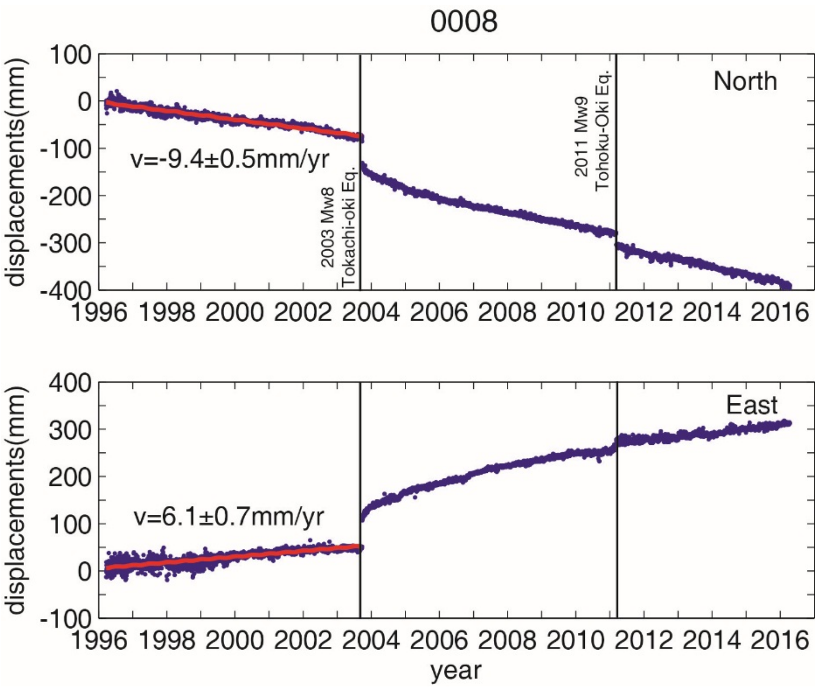
0008
North
East
v=-9.4±0.5mm/yr
v=6.1±0.7mm/yr
2003 Mw8 Tokachi-oki Eq.
2011 Mw9 Tohoku-Oki Eq.
displacements(mm)
year
1996 1998 2000 2002 2004 2006 2008 2010 2012 2014 2016

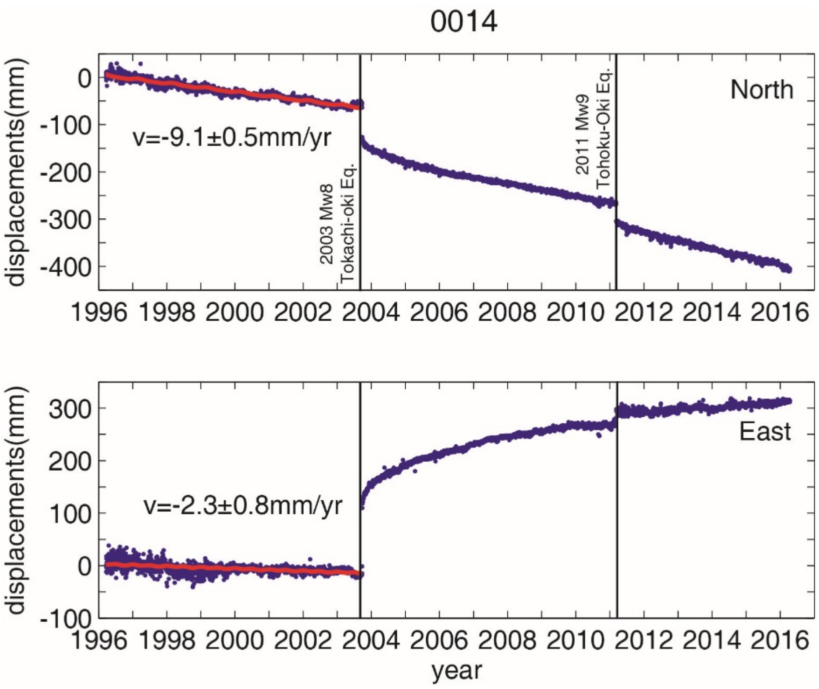
0014
North
East
v=-9.1±0.5mm/yr
v=-2.3±0.8mm/yr
2003 Mw8 Tokachi-oki Eq.
2011 Mw9 Tohoku-Oki Eq.
displacements(mm)
year
1996 1998 2000 2002 2004 2006 2008 2010 2012 2014 2016

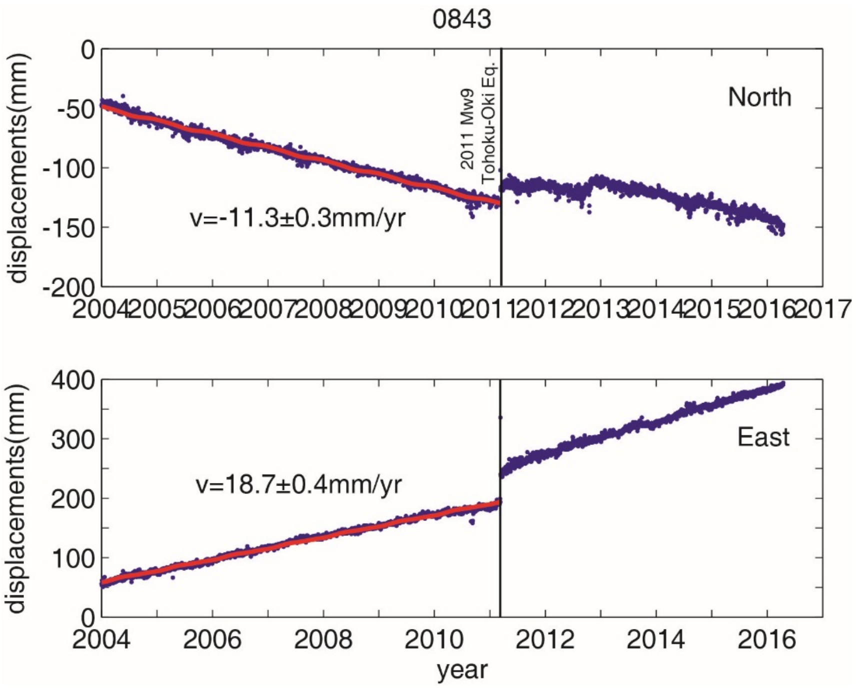
0843
North
East
v=-11.3±0.3mm/yr
v=18.7±0.4mm/yr
2011 Mw9
Tohoku-Oki Eq.
displacements(mm)
year
0
-50
-100
-150
-200
2004 2005 2006 2007 2008 2009 2010 2011 2012 2013 2014 2015 2016 2017
400
300
200
100
0
2004 2006 2008 2010 2012 2014 2016

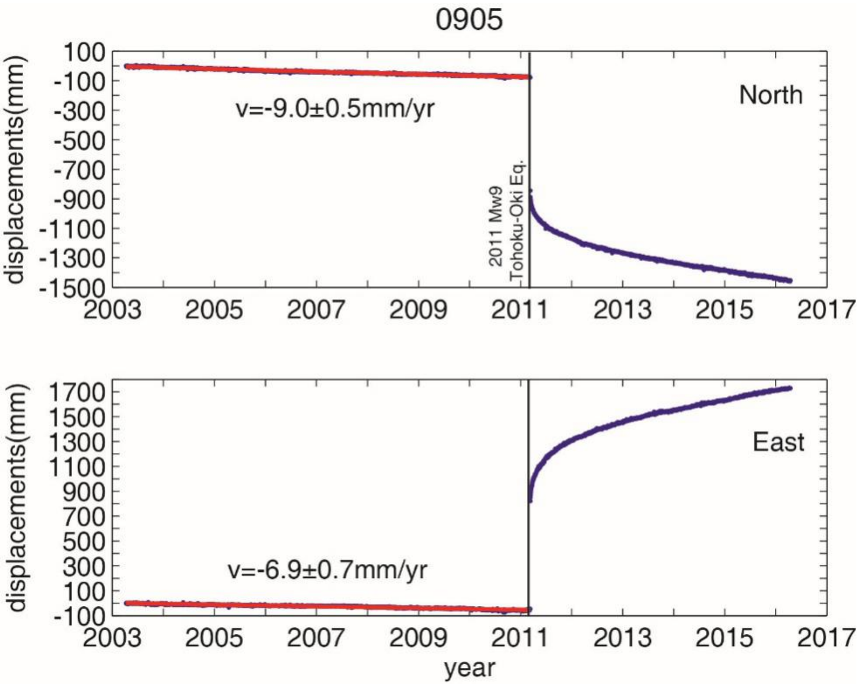
0905
North
East
v=-9.0±0.5mm/yr
v=-6.9±0.7mm/yr
2011 Mw9
Tohoku-Oki Eq.
displacements(mm)
year
100
-100
-300
-500
-700
-900
-1100
-1300
-1500
1700
1500
1300
1100
900
700
500
300
100
-100
2003 2005 2007 2009 2011 2013 2015 2017

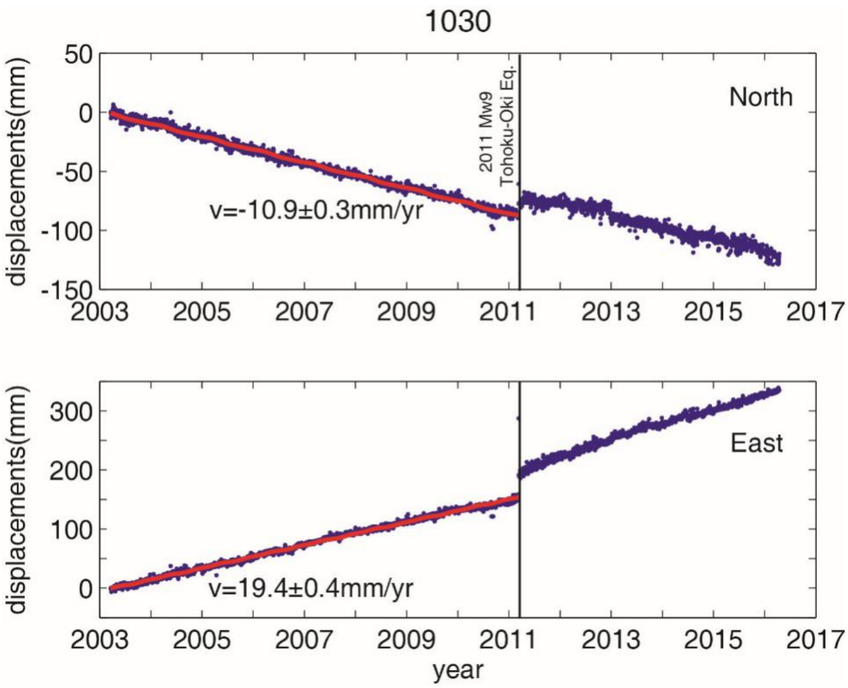
1030
North
2011 Mw9 Tohoku-Oki Eq.
v=-10.9±0.3mm/yr
displacements(mm)
50
0
-50
-100
-150
2003
2005
2007
2009
2011
2013
2015
2017
East
v=19.4±0.4mm/yr
displacements(mm)
300
200
100
0
2003
2005
2007
2009
2011
2013
2015
2017
year

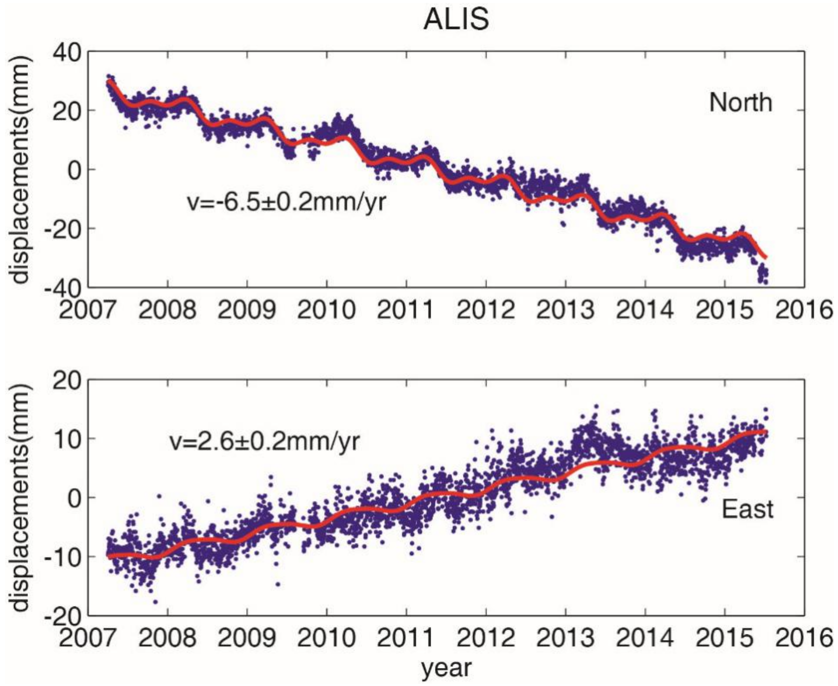
ALIS
North
v=-6.5±0.2mm/yr
displacements(mm)
40
20
0
-20
-40
2007
2008
2009
2010
2011
2012
2013
2014
2015
2016
v=2.6±0.2mm/yr
East
displacements(mm)
20
10
0
-10
-20
2007
2008
2009
2010
2011
2012
2013
2014
2015
2016
year

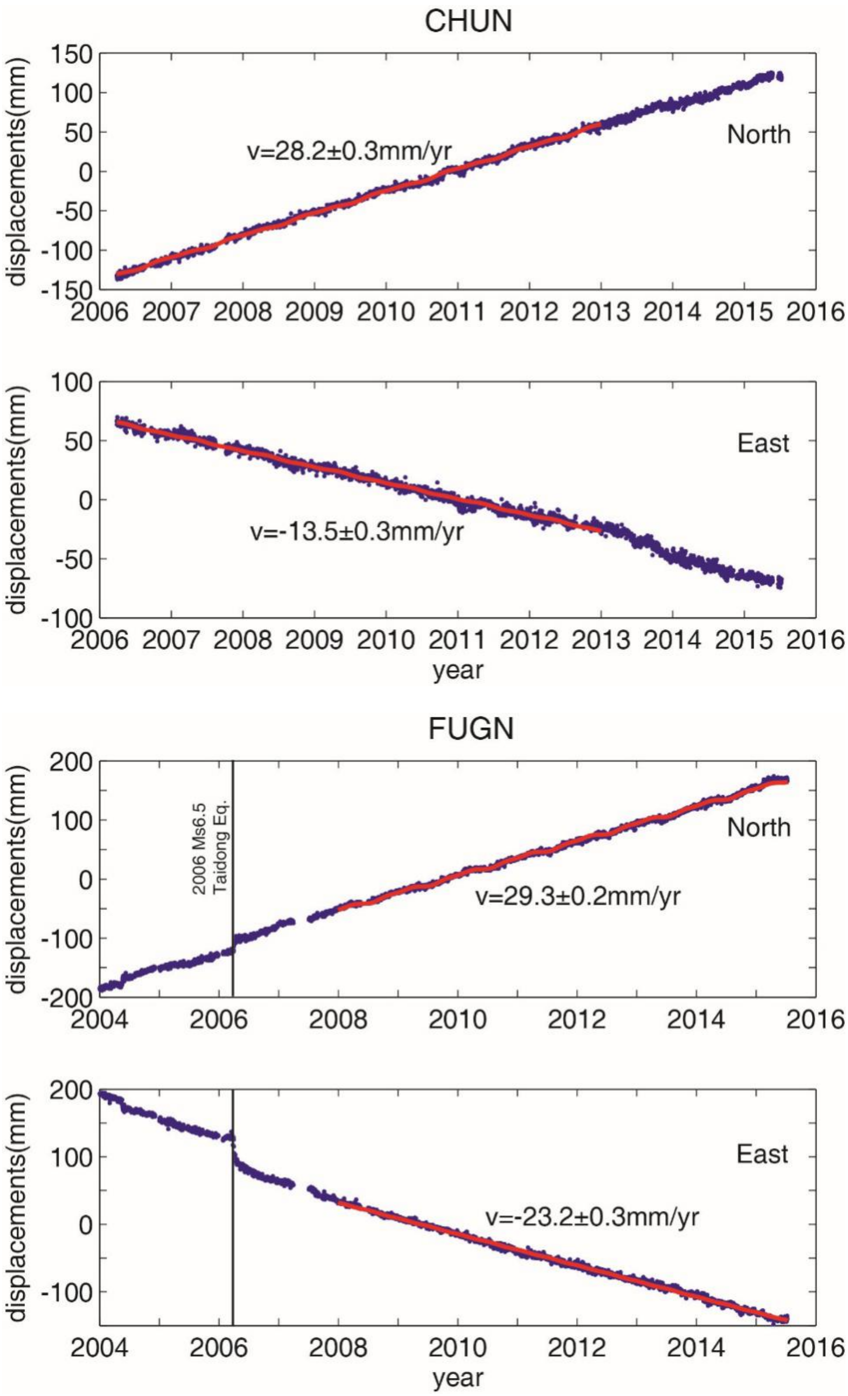
CHUN
displacements(mm)
North
v=28.2±0.3mm/yr
East
v=-13.5±0.3mm/yr
year
FUGN
2006 Ms6.5 Taidong Eq.
North
v=29.3±0.2mm/yr
East
v=-23.2±0.3mm/yr
year

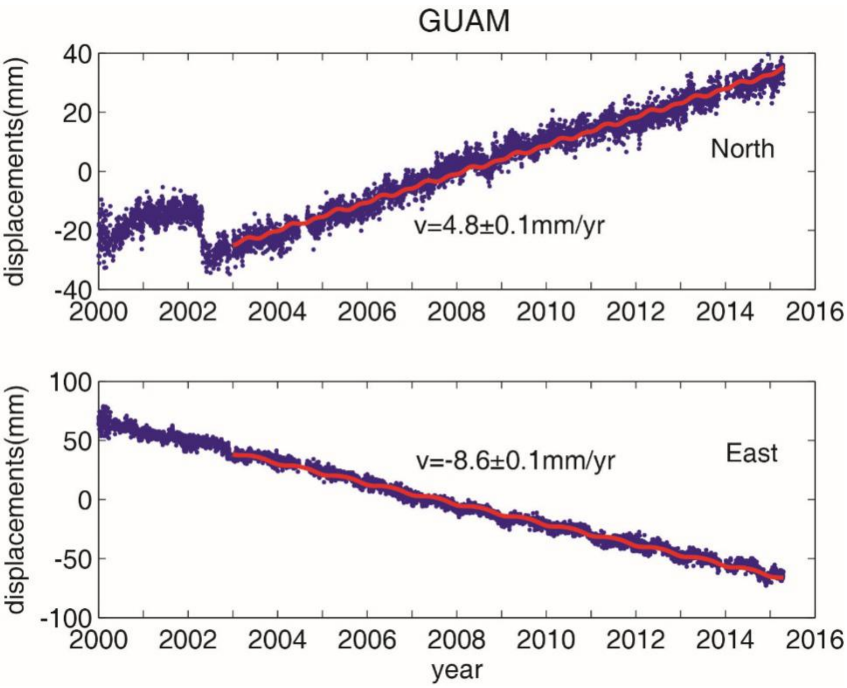

Figure S3. GPS time series fit by linear and seasonal variations (red curves).

A4. Estimation of Euler pole of the South China block

Figure S4 illustrates the corresponding site residuals of the South China block rotation, and the mean residuals for east and north components are 0.6 and 0.5 mm/yr, respectively. Tables S1 lists the rotation pole in two forms of expression.

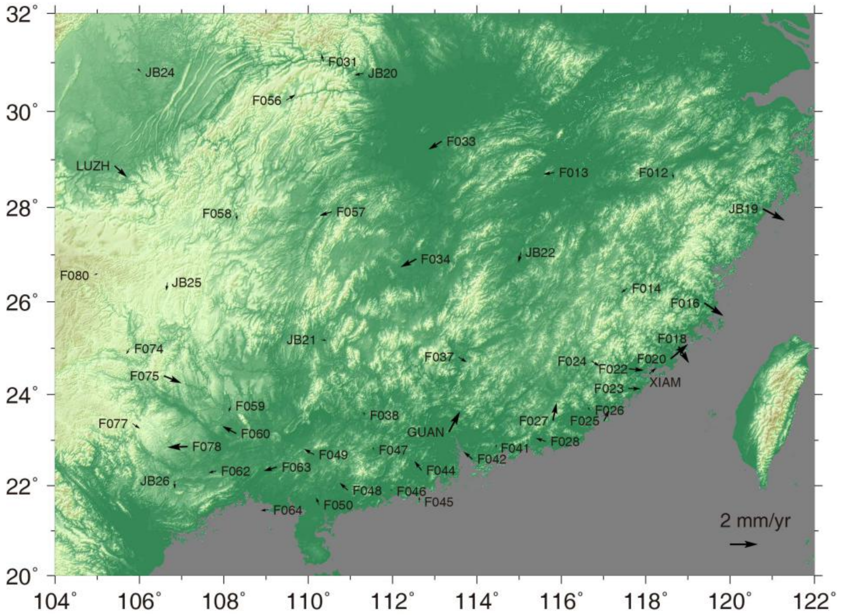

Figure S4. Post-fit residuals of the South China block estimation

Tables S1 The South China block rotation pole

ω_x	ω_y	ω_z	φ	λ	ω

		mas/yr			deg	deg/Ma.yr
	-0.128	-0.655	0.899	53.420	-101.102	0.311
±	0.012	0.029	0.015	1.275	1.139	0.006

A5. Slip rates on major faults in the Himalayan range

Block model constrained by GPS velocities is used to inverse slip rates on major faults in the Himalayan range.

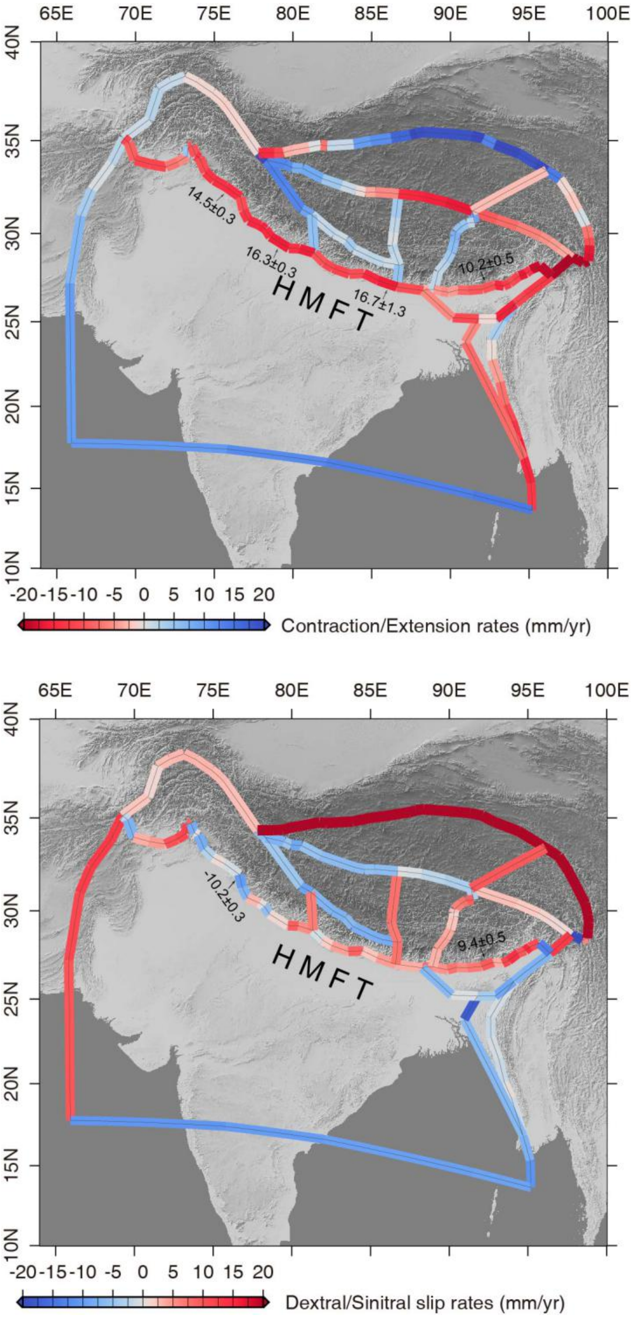

Figure S5. Slip rates on major faults

A6. GPS velocities and errors

GPS velocities and errors in East Asia with respect to the South China block (Ve_SC and Vn_SC), Eurasian plate (Ve_Eura and Vn_Eura) and ITRF2008 (Ve_ITRF2008 and Vn_ITRF2008) are provided as a separate file named “EastAsia_rates.xls”.

References

Chlieh, M., Avouac, J. P., Hjorleifsdottir, V., Song, T. R. A., et al. (2007). Coseismic slip and afterslip of the great mw 9.15 sumatra-andaman earthquake of 2004. Bulletin of the Seismological Society of America, 97(1A), S152-S173.

Fu, G. and Sun, W. (2010). Surface coseismic gravity changes caused by dislocations in a 3-D heterogeneous earth. Geophysical Journal of the Royal Astronomical Society, 172(2), 479-503.

Konca, A. O., Hjorleifsdottir, V., Song, T. R. A., et al. (2007). Rupture kinematics of the 2005 M_w 8.6 Nias－Simeulue earthquake from the joint inversion of seismic and geodetic data. Bulletin of the Seismological Society of America, 97(1A), S307-S322.

Wan Y. G., Shen Z. K., Wang M., et al. (2008). Coseismic slip distribution of the 2001 Kunlun Mountain pass west earthquake constrained by GPS and InSAR data. Chinese Journal of Geophysics, 51(4), 753-764.
